# Extended pluripotent stem cells facilitate mouse model generation

**DOI:** 10.1007/s13238-018-0573-0

**Published:** 2018-08-21

**Authors:** Guanghai Xiang, Haoyi Wang

**Affiliations:** 1 State Key Laboratory of Stem Cell and Reproductive Biology, Institute of Zoology, Chinese Academy of Sciences, Beijing, 100101 China; 2 University of Chinese Academy of Sciences, Beijing, 100049 China

Mouse embryonic stem (mES) cells, established in 1981 (Evans and Kaufman, 1981; Martin, 1981), were derived from the inner cell mass (ICM) of blastocysts and can be expanded *in vitro* for many passages, maintaining normal karyotype and differentiation potential. Upon introduction into blastocysts, mES cells can differentiate into all three germ layers, contributing to all the somatic lineages and germline. In 1998, James Thompson derived human embryonic stem (hES) cells from the ICM of human blastocysts (Thomson et al., 1998). Intriguingly, hES cells have many characteristics different from mES cells, including morphology and signaling pathway maintaining pluripotency (Burdon et al., 2002; Forsyth et al., 2002; James et al., 2005). In 2007, mouse epiblast stem cells (EpiSCs) were derived from the epiblast of post-implantation mouse embryo (Brons et al., 2007; Tesar et al., 2007). These mouse EpiSCs have distinct molecular and functional properties from mouse ES cells, while resemble human ES cells in many ways. Mouse ES cells and EpiSCs represent the *in vitro* counterpart of preimplantation and postimplantation epiblast, and these two phases were defined as naïve and primed pluripotency respectively (Nichols and Smith, 2009). The traditional human ES cells are similar to mouse EpiSCs as a primed pluripotent state. Recently, several groups described culture conditions to induce and maintain human ES cells at a naïve-like state (Chan et al., 2013; Duggal et al., 2015; Gafni et al., 2013; Takashima et al., 2014; Theunissen et al., 2014; Valamehr et al., 2014; Ware et al., 2014), suggesting that human pluripotent stem cells also have these two phases.

Although pluripotent stem cells can differentiate into all the cell types in an adult organism, neither naïve ES cells nor EpiSCs could contribute to extra-embryonic (ExEm) tissues, which mediate uterine implantation and subsequent maternal nutrition of the growing embryo and fetus (Beddington and Robertson, 1989). In 2017, two groups reported the derivation of extended (or expanded) pluripotent stem (EPS) cells, which could generate both embryonic and extra-embryonic lineages *in vivo* (Yang et al., 2017a, b). EPS cells could be efficiently derived from early embryos and through reprogramming, both in human and mouse. Remarkably, one single EPS cell injected into eight-cell embryo could contribute to both the embryo proper and the trophectoderm lineages. Single-cell transcriptome analysis revealed enrichment for blastomere-specific signature in EPS cells.

One of the most important applications of mES cells is to generate knockout mice. In this issue of *Protein* & *Cell*, two papers from Deng lab (Du et al., 2018; Li et al., 2018) showed that, compared to mES cells, EPS cells have superior advantages in generating mouse models. Li et al. showed that EPS cells had genetic and epigenetic stability better than ES cells after long-term culturing. When single EPS or ES cell was injected into eight cell embryos, EPS cells showed much better chimeric contribution capability. They further knocked human *IL3* and *IL6* genes into mouse endogenous loci using CRISPR-Cas9. After injecting these engineered EPS cells into tetraploid embryos, they were able to derive *IL3* and *IL6* knock-in mice directly with an efficiency of one mouse out of ten injected embryos, while injecting ES cells failed to obtain any live born. These results showed that gene targeting in mouse EPS cells combined with tetraploid complementation (Nagy et al., 1993) can efficiently produce mouse models in approximately 2–3 months.

Since only a few mouse strains are permissive for ES cells derivation, Du and colleagues attempted to derive EPS cells from non-permissive NOD-*scid Il2rg*^−/−^ strain. They successfully established EPS cells from NOD-*scid Il2rg*^−/−^ stain via two methods: *de novo* derivation from blastocysts and chemical reprogramming from embryonic fibroblasts. *In vitro* long-term culture showed these EPS cells kept normal karyotypes, and contributed to both ICM and trophectoderm lineages when injected into embryos. They also showed that gene targeting worked efficiently in EPS cells derived from NOD-*scid Il2rg*^−/−^ background.

Genetically modified mouse models are invaluable tools for biology and biomedical research. These two studies raised some exciting opportunities for improving mouse model generation. Although CRISPR-Cas9 based gene editing in zygote could generate knockout and knock-in mice efficiently (Wang et al., 2013; Yang et al., 2013), more sophisticated manipulation such as large transgene knock-in and conditional allele generation could still be easier using gene targeting in pluripotent stem cells. With superior genetic and epigenetic stability and efficient tetraploid complement capability, EPS cells could serve as a very useful system to generate genetically modified mouse models. With the help of CRISPR-Cas9, multiple sophisticated genetic modifications can be engineered in EPS cells and then mouse will be derived directly from these cells via tetraploid complementation.

Since NOD-*scid Il2rg*^−/−^ strain is highly immunodeficient, it is widely used for generating humanized mouse models, such as patient derived xenograft (PDX) model. It will be interesting to know whether the results of these two studies can be combined to generate human *IL6* knock-in NOD-*scid Il2rg*^−/−^ mouse through tetraploid complementation. If this is successful, it sure will facilitate the generation of more sophisticated models on this important strain background.

Although EPS cells have developmental potency to contribute to both embryonic and extra-embryonic lineages, they are still not bona fide totipotent (Jaenisch et al., 2018). As Li and colleagues showed, when one single EPS cell was injected into eight cell embryos, about 30% of the E10.5 embryos have more than 50% cells coming from this single injected EPS cell. This suggests that EPS cells have better development potential than natural blastomere! Upon further development of culture condition, whether EPS cells alone can contribute to the entire embryos is an extremely exciting question to ask.

So far EPS cells have only been derived from human and a few mouse strains, another very exciting future direction is to derive EPS cells from more species, especially in livestock animals that were non-permissive to pluripotent stem cell establishment. If successful, this will greatly improve the genetic modification and production of other species such as non-human primate and livestock.

## Notes

This work was supported by the National Natural Science Foundation of China (No. 31471215), the Strategic Priority Research Program of Chinese Academy of Sciences (No. XDA16010205), and the National Key Research and Development Program of China (No. 2016YFA0101402).

The authors declare that they have no conflict of interest.
